# Can We Provide Safe Training and Competition for All Athletes? From Mobile Heart Monitoring to Side Effects of Performance-Enhancing Drugs and MicroRNA Research

**DOI:** 10.3390/diagnostics11030492

**Published:** 2021-03-10

**Authors:** Łukasz A. Małek, Marek Postuła

**Affiliations:** 1 Department of Epidemiology, Cardiovascular Disease Prevention and Health Promotion, National Institute of Cardiology, 04-635 Warsaw, Poland; 2 Department of Experimental and Clinical Pharmacology, Medical University of Warsaw, 02-097 Warsaw, Poland; mpostula@wum.edu.pl

The foundations of sports cardiology include promoting physical activity and an ability to provide a safe environment for training and competition for all athletes at all levels, from professional to recreational. To combine these two aims, reliable tools to perform pre-participation screening are needed. Moreover, those at high risk of potentially life-threatening events should be advised to limit their training load, while others should be reassured that there is no exercise-related cardiovascular risk. We currently observe an advent of new portable devices for remote and mobile heart monitoring and several new, promising biochemical markers, which can support athletes’ diagnostic processes. In this Special Issue of the *Diagnostics* journal entitled “Diagnostic Challenges in Sports Cardiology”, we present a series of 13 manuscripts, including eight original works, three reviews, and two case reports, which give a glimpse of the current research topics in the area of sports cardiology.

An excellent example of the balance between the benefits and safety of continued training is a presented case of a 20-year-old athlete with exertional syncope found to harbor a rare genetic disorder causing catecholaminergic polymorphic ventricular tachycardia (CPVT) and predisposing him to severe ventricular arrhythmias and risk of sudden cardiac death [1]. Prompt diagnosis, followed by administration of pharmacological treatment and implantation of a cardiac defibrillator (ICD), permitted the athlete to return to moderate physical activity without clinical events for the time being. This would not have been possible if not for the previous observational reports demonstrating that continued sport participation in such patients might be considered on an individual basis [2]. The current state of knowledge on management and decision-making in several cardiac conditions in athletes should be based on separate algorithms from the non-athletic population and on an individual basis. An example of such an approach concerning the presence of an asymptomatic pre-excitation in junior athletes is demonstrated and discussed in a review of the literature performed by Książczyk et al. [3].

To increase confidence regarding athletes’ diagnostic and management decisions, it is crucial to perform differential diagnosis between physiological adaptation to training (namely considering the athlete’s heart) and pathological changes. Adaptation to physical activity has been demonstrated in recent decades, mainly in professional athletes, while amateurs, veterans, and other special groups are only now beginning to increasingly enter the scope of research [4]. Interestingly, there may be different athletic pictures of the athlete's heart related to diet, even within the same sports category. Król et al. describe differences between amateur vegan and omnivorous middle-age athletes’ hearts. They found a larger left ventricular chamber and signs of better systolic and diastolic function of the left ventricular muscle in the first group, with these changes considered to be positive [5]. Moreover, vegans demonstrated a higher peak oxygen uptake, a marker of physical fitness. On the other hand, an athlete’s heart's features may be almost absent in an elite middle-age ultra-marathoner competing at the highest level, as Gajda et al. have demonstrated [6].

Another aspect of sports cardiology is the continuous monitoring of athletes at different time points throughout their careers and during a single season. Doing so may help to detect markers of a new-onset disease, fatigue, and overtraining or predict physical fitness, as described by several articles in this issue. Gajda presents the results of a survey performed on 100 amateur endurance athletes, ten coaches, and ten sports doctors on the importance of heart rate monitoring (HRM) and suggests improvements to make these devices’ application more useful [7]. All participants preferred optical rather than strap devices and perceived the possibility of continuous electrocardiogram (ECG) monitoring during training to improve athletes’ safety. The manuscript contains a review of new devices used for HRM in athletes and is accompanied by a case report showing how HRM has been applied to detect atrioventricular nodal reentrant tachycardia (AVNRT) over six years in an amateur triathlete unwilling to undergo ablation [8]. Grzebisz looked for determinants of cardiovascular capacity in a group of 16 amateur long-distance skiers before the start of the preparation period. She assessed several morphological and biochemical markers, of which percentage of monocytes, the concentration of sodium, and total calcium were found as predictors of peak oxygen uptake in the regression model [9]. In a series of two articles, Gąsior & Hoffmann et al. focus on the analysis of heart rate (HR), respiratory rate, and heart rate variability (HRV) parameters as potential markers of fatigue and overtraining [10,11]. For that purpose, they studied a group of 12 elite modern pentathlonists and described baseline responses of the studied parameters to sympathetic nervous system activity stimulation, suggesting that the analysis should be individualized [10]. They also found that HRV should be interpreted concerning concomitant differences in HR and respiratory rate and technique of registration [11].

It is still undetermined whether participation in ultra-endurance exercises and competitions leads to acute and prolonged cardiac injury [12]. In this issue, in a group of 18 amateur middle-aged and veteran athletes, we demonstrate that participation in a 100-km running event caused only a mild increase in troponin T, which was not necessarily linked to cardiac injury, as demonstrated by the correlation of troponin T increase with markers of inflammation and lactic acid concentration during the race [13]. This is in line with findings of a detailed cardiac (ECG, echocardiography, cardiac magnetic resonance with resonance spectroscopy) and biochemical panel analysis performed before, 1–2 days after, and ten days after the race in a winner of a 24-h ultra-endurance run [6]. Cardiac imaging did not disclose any alterations, and there were only transient laboratory changes, most likely reflecting muscle damage, liver cell damage, activation of inflammatory processes, effects on the coagulation system, exercise-associated hyponatremia, and cytoprotective or growth-regulatory effects.

Safe sport is dependent on the intended use of legal performance-enhancing medications and the avoidance of illegal substances, which often affect the heart. For that purpose, Sivalokanathan et al. review current data on the cardiac effects of the most commonly used legal and illegal substances (caffeine and anabolic steroids) [14]. A final manuscript of that issue gives a glimpse into the potential future of sports cardiology. Soplińska et al. present a comprehensive review on the role of microRNAs (small particles that regulate the post-transcription gene expression) as biomarkers of systemic changes in response to endurance training [15]. The authors found that miR-1, miR-133, miR-21, and miR-155 are crucial in adaptive response to exercise. It will be interesting to see what the future holds for these markers.
